# Supplementary material for: Protein Interactomic Analysis of SAPKs and ABA-Inducible bZIPs Revealed Key Roles of SAPK10 in Rice Flowering
Source: Int J Mol Sci. 2019 Mar 21;20(6):1427. doi: 10.3390/ijms20061427 (PMC6471077; doi:10.3390/ijms20061427)
Supplement: Supplementary file 1 [file ijms-20-01427-s001.pdf]

Table S1. Primers used in this study

Name	Primer(5'--------3')	Usage
bZIP10 F	CACCATGGCATCGGAGATGAGCAA	Yeast-two-hybrid
bZIP10 R	TCACCACATGCAGCTGCCGC	
bZIP11 F	CACCATGGTGCAAGGTGAGGAGAG	
bZIP11 R	TCAAAATCCTGAGTACTGAT	
bZIP35 F	CACCATGGAGGGTGAACCCTCACG	
bZIP35 R	TTATTGTGGCTGATTATGCT	
bZIP52 F	CACCATGATGAAGAAGTGCCCGTC	
bZIP52 R	TTAAGGCCACACATCAGCCG	
bZIP55 F	CACCATGGCGAACTACCACCACCA	
bZIP55 R	TCAGAATTGAGTTGATGAAG	
bZIP71 F	CACCATGTCGAGTGGGACCTCGTC	
bZIP71 R	TCAGAAGCACTGGTACTGGT	
bZIP75 F	CACCATGAACAGGGAGAAATCCCC	
bZIP75 R	TCAGTTGCCGCTGCTTCCTG	
bZIP77 F	CACCATGGCGATGGAGGACGACGA	
bZIP77 R	TCAGAATGGCGCGGAGAGCA	
bZIP83 F	CACCATGGGAGAGGCTAGGAGAGG	
bZIP83 R	TCAGAAAGCTGAAAATTGGC	
SAPK1 F	CACCATGGAGCGGTACGAGGTGAT	
SAPK1 R	TCACAAGGCGCACACGAAGTC	
SAPK2 F	CACCATGGAGAGGTACGAGGTGAT	
SAPK2 R	TCACAATGCGCACACGAAGTC	
SAPK3 F	CACCATGGAGGAGAGGTACGAGGC	
SAPK3 R	TCAGTAGGTGTCATACTCATC	
SAPK4 F	CACCATGGAGAAGTACGAGGCGGT	
SAPK4 R	TCATATGCGCAGTGAGCTCATA	
SAPK5 F	CACCATGGAGAAATACGAGCCAGT	
SAPK5 R	TCAGGAGATTTGGAGGTTTGA	
SAPK6 F	CACCATGGAGAAGTACGAGCTGCT	
SAPK6 R	TCAGCTCTTCTGCAAGTCACAG	
SAPK7 F	CACCATGGAGAGGTACGAGCTGCT	
SAPK7 R	TCAGCTGAGCTGAAACTCACC	
SAPK8 F	CACCATGGCAGCGGCGGGGGCCGG	
SAPK8 R	TTACATCGCATAGACGATC	
SAPK9 F	CACCATGGAGAGGGCGGCGGCGGG	
SAPK9 R	TTACATGGCATATACGATCTC	
SAPK10 F	CACCATGGACCGGGCGGCGCTGAC	
SAPK10 R	TCACATAGCGTATACTATCT	
qRT-bZIP10 F	GGACGCAACTTTGGTTCCAT	qRT-PCR
qRT-bZIP10 R	GTCTACCACCTTGGCTTCCT	

qRT-bZIP11 F	GAGGAAGAGCAGGCTGAGAA	
qRT-bZIP11 R	GACCGTGCTCTTTGGAGTTC	
qRT-bZIP35 F	CACCATCCTCAACCTCACCT	
qRT-bZIP35 R	GAAGGTGGTGGTTCCGAGTA	
qRT-bZIP52 F	GTGGCACGAAATCCACAGAA	
qRT-bZIP52 R	CCTTTGAGTTGCTCGACCTG	
qRT-bZIP55 F	GAACTACCACCACCAGGAGT	
qRT-bZIP55 R	AGATGGAGAAGAGGGAGCTG	
qRT-bZIP71 F	ACAACACCAGTGCTACTCCT	
qRT-bZIP71 R	AGTCTATGGGTGGCTGGTTC	
qRT-bZIP75 F	GCCATTGCACCCTCTACAAG	
qRT-bZIP75 R	CTGCTTCCTGACTTGCCATC	
qRT-bZIP77 F	GCAAGAGGCGGATGATCAAG	
qRT-bZIP77 R	CTCGGTCTCCAGGTTGTTGA	
qRT-bZIP83 F	ACGTGTTCCACCTCATCACT	
qRT-bZIP83 R	CAGATCGGACGGTTGGAAAC	
qRT-SAPK1 F	GAACCACCGATCACTGAAGC	
qRT-SAPK1 R	CACTGAATCTCCCTGCGTTG	
qRT-SAPK2 F	AACCACCCATGGTTCCTCAA	
qRT-SAPK2 R	CTGGACGCTCATCTGGTACT	
qRT-SAPK3 F	GAACACCCTCCTGGATGGAA	
qRT-SAPK3 R	TCACGTACAGTGTCACACCA	
qRT-SAPK4 F	GGCCACCGGATAGATGAGAA	
qRT-SAPK4 R	GCTGCGAACTCCATGACAAT	
qRT-SAPK5 F	AAGATCTGCGACTTCGGCTA	
qRT-SAPK5 R	GAGCATGACGTAGAGGGTGA	
qRT-SAPK6 F	AAAGACACCACCTCCATCGT	
qRT-SAPK6 R	GTCGGGATTCTTACCGTCCT	
qRT-SAPK7 F	TCCATGTATCCCAGGACTGC	
qRT-SAPK7 R	AGCTCTCTTGGCAGGTTCTT	
qRT-SAPK8 F	GCAAGTATGCCACCGTGATT	
qRT-SAPK8 R	TCAGCAGTCTTGCCATCGTA	
qRT-SAPK9 F	TGTTGGCAACCCAGCTAGTA	
qRT-SAPK9 R	TGCTGCTGGAATTGTTGCTT	
qRT-SAPK10 F	TCGCCTCAAGATATGCGACT	
qRT-SAPK10 R	CACGACCACACATCAGCAAT	
qMADS 15F	GCCACCTTATGCTTGAGTCC	
qMADS 15R	TTCTTCTGCCTCTCCACCAG	
GST-SAPK10 F	TGGATCCCCGGAATTCATGGACCGGGCGGCGCTGACGG	Pull-Down/Phosphorylation
GST-SAPK10 R	GATGCGGCCGCTCGAGCCATAGCGTATACTATCTCCCCA	
GST-SAPK9 F	TGGATCCCCGGAATTCATGGAGAGGGCGGCGGCG	
GST-SAPK9 R	GATGCGGCCGCTCGAGCATGGCATATACGATCTCTCCGC	
HIS-bZIP77 F	CGGAATTCATGGCGATGGAGGACGA	

HIS-bZIP77 R	CCAAGCTTTCAGAATGGCGCGGAGAGCA	
OxSAPK10 F	GCTCTAGAATGGACCGGGCGGCGCTGACG	Ox-SAPK10
OxSAPK10 R	TGCACTGCAGTCACATAGCGTATACTATCT	

Figure S1. Kinase assay of SAPK10 on bZIP77.

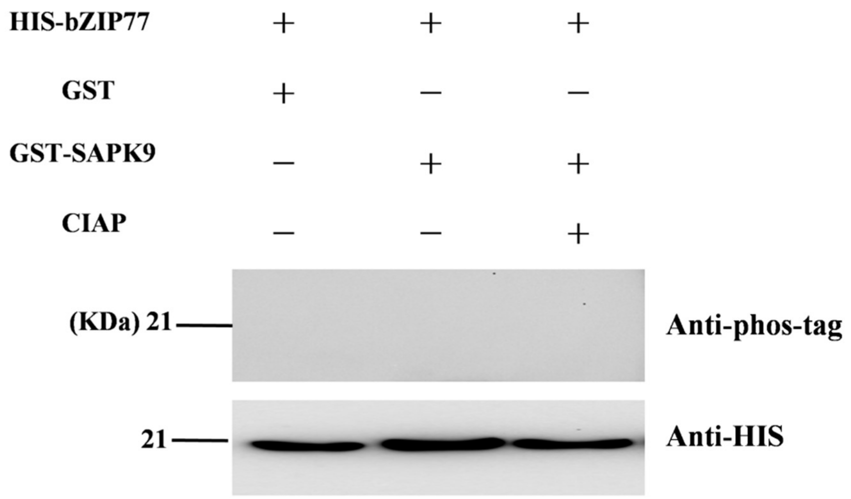

Figure S2. The transcriptional level and heading date of SAPK10 RNAi lines.

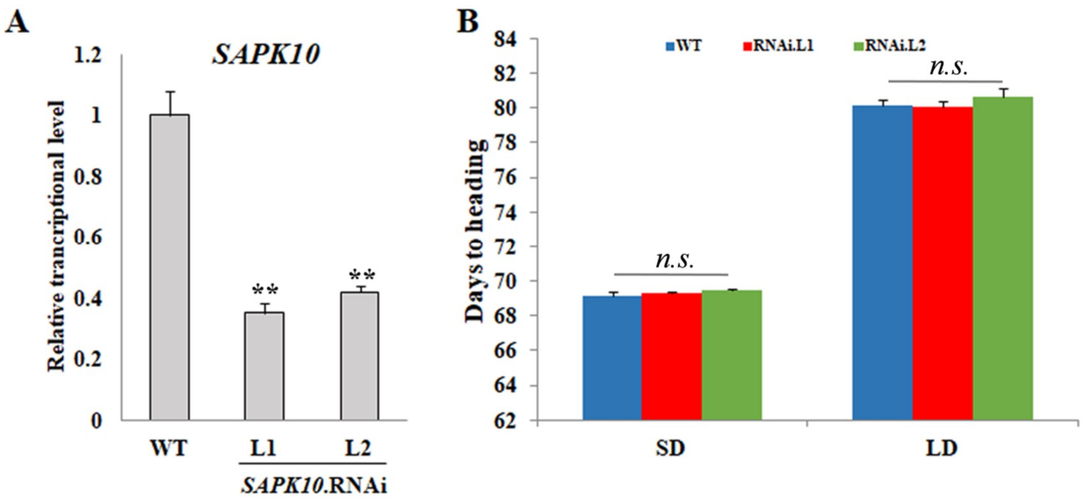

(A) Transcriptional levels of two representative *SAPK10* RNAi lines. Three technical replicates were conducted. Data is presented as mean±SD (n=3), ** indicates P<0.01 by *students'* t-test. (B) Quantifications of the heading date of SAPK10 RNAi lines under LD and SD conditions (n=5). N.S. indicates no statistically significant differences by *students'* t-test.
